# Systematic review of clinical prediction models to support the diagnosis of asthma in primary care

**DOI:** 10.1038/s41533-019-0132-z

**Published:** 2019-05-09

**Authors:** Luke Daines, Susannah McLean, Audrey Buelo, Steff Lewis, Aziz Sheikh, Hilary Pinnock

**Affiliations:** 1 Asthma UK Centre for Applied Research, Usher Institute of Population Health Sciences and Informatics, The University of Edinburgh, Edinburgh, UK; 2 Scottish Collaboration for Public Health Research and Policy, The University of Edinburgh, Edinburgh, UK; 3 Edinburgh Clinical Trials Unit, Usher Institute of Population Health Sciences and Informatics, The University of Edinburgh, Edinburgh, UK

**Keywords:** Asthma, Diagnosis, Respiratory signs and symptoms

## Abstract

Diagnosing asthma is challenging. Misdiagnosis can lead to untreated symptoms, incorrect treatment and avoidable deaths. The best combination of clinical features and tests to achieve a diagnosis of asthma is unclear. As asthma is usually diagnosed in non-specialist settings, a clinical prediction model to aid the assessment of the probability of asthma in primary care may improve diagnostic accuracy. We aimed to identify and describe existing prediction models to support the diagnosis of asthma in children and adults in primary care. We searched Medline, Embase, CINAHL, TRIP and US National Guidelines Clearinghouse databases from 1 January 1990 to 23 November 17. We included prediction models designed for use in primary care or equivalent settings to aid the diagnostic decision-making of clinicians assessing patients with symptoms suggesting asthma. Two reviewers independently screened titles, abstracts and full texts for eligibility, extracted data and assessed risk of bias. From 13,798 records, 53 full-text articles were reviewed. We included seven modelling studies; all were at high risk of bias. Model performance varied, and the area under the receiving operating characteristic curve ranged from 0.61 to 0.82. Patient-reported wheeze, symptom variability and history of allergy or allergic rhinitis were associated with asthma. In conclusion, clinical prediction models may support the diagnosis of asthma in primary care, but existing models are at high risk of bias and thus unreliable for informing practice. Future studies should adhere to recognised standards, conduct model validation and include a broader range of clinical data to derive a prediction model of value for clinicians.

## Introduction

Making an accurate diagnosis of asthma is fundamental to improving asthma care and outcomes. However, asthma is commonly misdiagnosed, with over- and under-diagnosis of asthma in children and adults reported.^1–3^ Over-diagnosis leads to costly, potentially harmful treatment and unnecessary health care, whilst under-diagnosis risks inadequate treatment and avoidable morbidity and mortality.

Accurately diagnosing asthma is challenging. Asthma is a heterogeneous disease comprising different genotypes, endotypes and phenotypes.^4^ There is no ‘gold’ reference standard that can categorically confirm or refute the diagnosis. Asthma is thus a clinical diagnosis, but individual symptoms, signs and tests have poor sensitivity/specificity for the diagnosis. Uncertainty about the best combination of clinical features and tests for asthma diagnosis is reflected in conflicting recommendations between national^5,6^ and international^7^ guidelines and highlighted in commentaries seeking to reduce confusion for clinicians.^8,9^

One solution could be to use a clinical prediction model, a data-driven algorithm that combines at least two predictors, such as elements from a clinical history, physical examination, test results and/or response to treatment, to estimate the probability that an outcome is present.^10^ Clinical prediction models can assist healthcare professionals to weigh up the probability of a diagnosis, enhance shared decision-making and aid patient stratification into subtypes.^11,12^ As most asthma diagnoses occur in non-specialist settings,^4^ where health problems typically present in an undifferentiated manner, and assessment is often based on probability,^13^ a prediction model could increase the accuracy of asthma diagnosis by supporting the appraisal of available clinical information and guiding next steps.

We aimed to identify, compare and synthesise existing clinical prediction models designed to support the diagnosis of asthma in children and adults presenting with symptoms suggestive of asthma in primary care or equivalent settings.

## Results

### Study selection

**Fig. 1:**
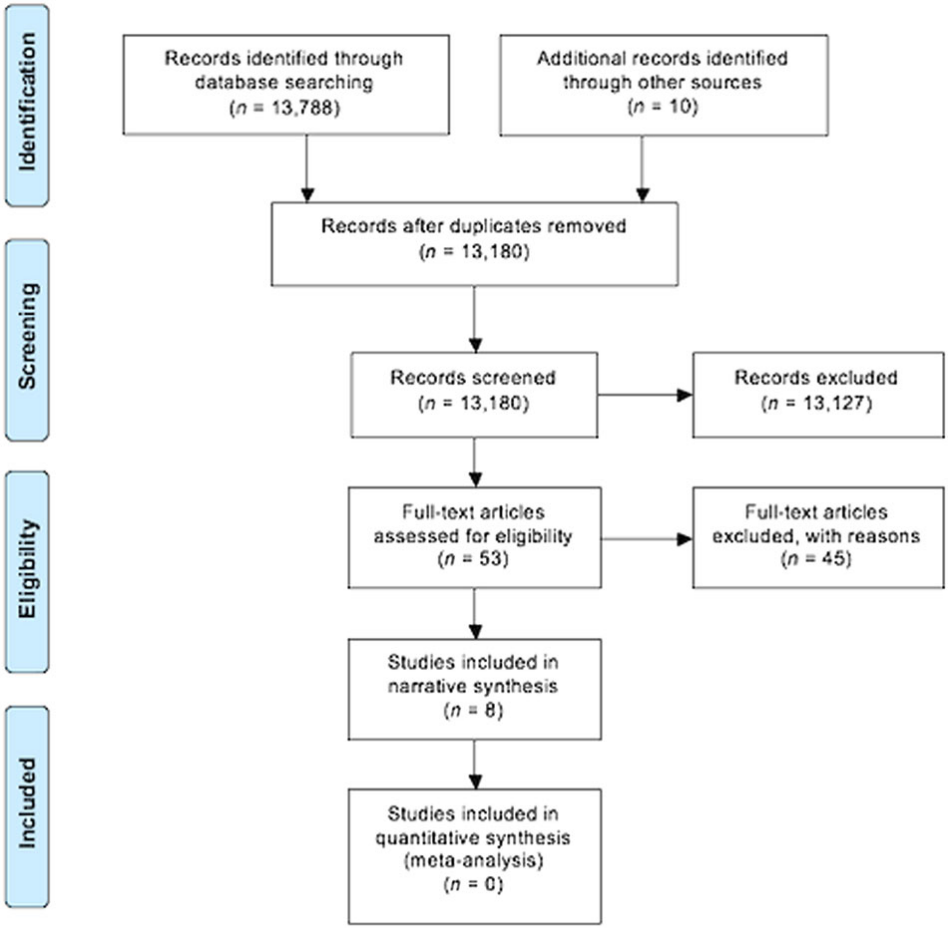
Preferred Reporting Items for Systematic Review and Meta-Analysis (PRISMA) flow diagram

Our searches identified 13,798 records. Following the removal of duplicates, 13,180 titles and abstracts were screened (Fig. 1). Fifty three articles were reviewed in full text, with 45 articles excluded (Supplementary Table 1). Eight articles from seven studies met the review criteria and were included.^14–21^

### Study characteristics

The included studies all derived new clinical prediction models (Table 1). Each study presented a model that could be used to aid the diagnosis of asthma; however, study rationale varied, and this was reflected in the design and approach to modelling used. Six studies used multivariable logistic regression to derive their prediction models.^14,15,17,18,20,21^ One study developed a decision tree.^19^ Six models were derived from adults,^14,17–21^ and one from children.^15^ The three studies^14,18,21^ that recruited exclusively from out-patient departments were conducted in countries without established primary care services, where patients commonly presented with undifferentiated symptoms to secondary care.^22,23^

**Table 1:** Characteristics of the included prediction modelling studies

Study ID Location Study design and dates	Source population, *N*	Inclusion criteria	Exclusion criteria	Included in CPM (% of *N*)	Outcome measurement	Outcome, number of events		No. of candidate predictor (EPV)^a^	CPM performance measures	Comments
Choi 2007 South Korea Cohort, NR	OPD 6 hospitals (*N* = 302)	Adults with ‘various respiratory symptoms’	NR	302 (100%)	Either: 1. +ve reversibility test OR 2. +ve bronchial provocation	Asthma No asthma	210 98	11 (8.9)	AUROC: 0.647±0.033	Prevalence of asthma in sample was 70%
Hall 2001 Connecticut, USA Prospective Cohort June 1998–May 1999	OPD, 4 clinics (*N* = 211)	Children (age NR) presented to an OPD (respiratory/ cardio/ endocrine/ general)	Children who had previously attended the respiratory OPD	178 (84%)	Healthcare provider decided. >2 episodes reversible airflow obstruction either 1. Symptoms resolved OR 2. Spirometry ‘if feasible’	Asthma No asthma	95 83	4 (20.8)	Any of four symptoms Sens: 100 (94–100) Spec: 55 (45–66)	Limited information regarding methods for primary care analyses
	6× Primary care (*N* = 4280)	Children (6 m to 18 y) presented to primary care for ‘any reason’	NR	3961 (93%)	NR	Asthma No asthma Other	1575 2063 323	4 (393.8)	NR	
Hirsch 2001 and 2004 Manchester, UK Cross-sectional, 1995	2× Primary care (*N* = 420)	Adults (≥16 y) who completed a postal survey	NR (individuals were selected to achieve an asthma-enriched sample based on responses to six questions)	180 (43%)	After clinical assessment, asthma confirmed if consensus of three experts was ≥50% probability of asthma	Asthma No asthma	84 96	15 (5.6)	NR	Different outcome measurements across analyses
Lim 2014 South Korea Cohort NR	OPD, 1 hospital (*N* = 680)	Adults (≥18 y) presented to OPD with respiratory symptoms suggesting asthma; dyspnoea, cough, tight chest, wheezing	1. Current LRT disease: pneumonia/emphysema/TB/other 2. ENT infection or disease 3. Respiratory admission (<3 m) 4. Active CVD/Ca/immune disease 5. Pregnant/breastfeeding	680 (100%)	Symptom questionnaire suggested asthma AND +ve bronchial provocation	Asthma No asthma	164 516	5 (32.8)	AUROC: 0.610 ±0.029	Baseline characteristics between asthma and non-asthma groups was comparable except BMI
Metting 2016 [Derivation] Groningen, Netherlands Retrospective cohort 2007–2012	Primary and secondary care interface (*N* = 10,058)	Adults (≥15 y) with respiratory complaints	Participants excluded if unable to perform spirometry	9297 (92%)	1 of 10 experienced respiratory physicians made diagnosis based on spirometry results and history	Asthma COPD ACOS Other	4125 1716 711 2745	22 (32.3)	Sens: 0.79 Spec: 0.75 PPV: 0.72 NPV: 0.82	Decision tree developed to distinguish asthma, COPD and ACOS
[External validation] Rotterdam, Netherlands Retrospective cohort 2008–2011	Primary and secondary care interface (*N* = 3142)	Referred by GP for diagnosis		3141 (100%)	One of four clinicians (two respiratory physicians and two GPs with specialist interest) made diagnosis as above	Asthma Prob asthma COPD ACOS Other	685 836 555 247 818	N/A	Sens: 0.78 Spec: 0.60 PPV: 0.35 NPV: 0.91	Two authors report conflict of interest
Schneider 2015 Heidelberg/Bavaria, Germany Prospective cohort Feb 2006–June 2007	10× Primary care 6× Private practices (*N* = 560)	Adults (age NR) first presentation with dyspnoea/cough/ expectoration >2 m	1. RTI <6 w of assessment 2. Past history: OAD, unstable CAD, arrhythmia, active hyperthyroidism 3. Pregnant	553 (99%)	Expert decision from history/exam and either: 1. +ve reversibility test OR 2. +ve bronchial provocation	Asthma COPD ACOS Other	229 30 8 286	16 (<10)^b^	GP data AUROC: 0.82 (0.75–0.89) Combined data AUROC: 0.75 (0.71–0.79)	Present three CPMs derived from GP, private practice and combined datasets
Tomita 2013 Osaka, Japan Prospective cohort Jan 2008–Sept 2011	OPD (*N* = 4129)	Adults (18–88 y) first presentation to OPD with respiratory symptoms	1. Pregnant/breastfeeding 2. LRT disease, for example, atelectasis/IPF/ pneumothorax/pneumonia/ bronchitis 3. Current ICS/OCS, ACEI, BB 4. Chest pain or haemosputum 5. Abnormalities on X-ray	556 (13%)	Expert decision from history and either: 1. +ve reversibility test OR 2. +ve bronchial provocation	Asthma No asthma	367 199	17 (11.7)	High probability of asthma: Score ≥3: Sens: 0.35 Spec: 0.92	If asthma was diagnosed at first visit, patients were started on ICS before bronchial provocation completed <8 weeks

*Sens* sensitivity, *Spec* specificity, *PPV* positive predictive value, *NPV* negative predictive value, *AUROC* area under the receiver operating characteristic curve, *NA* not applicable, *NR* not reported, *CPM* clinical prediction model, *OPD* out-patient department, *+* *ve* positive, *LRT* lower respiratory tract, *TB* tuberculosis, *ENT* ear nose and throat, *CVD* cardiovascular disease, *Ca* cancer, *GP* general practitioner, *COPD* chronic obstructive pulmonary disease, *ACOS* asthma COPD overlap syndrome, *RTI* respiratory tract infection, *OAD* obstructive airways disease, *CAD* coronary arterial disease, *IPF* idiopathic pulmonary fibrosis, *ICS* inhaled corticosteroid, *OCS* oral corticosteroid, *ACEI* angiotensin-converting enzyme inhibitor, BB beta blocker, BMI body mass index, *m* months, *w* weeks, *y* years

^a^*EPV* = Events per variable is calculated by dividing the number of events by the number of candidate predictors (prior to selection)

^b^Schneider (2015) stated that the events per variable was <10

### Risk of bias

All included studies were judged to be at high risk of bias. Bias was introduced by various means, though certain limitations were shared by several studies (Table 2). Most notable was the lack of model validation. See Supplementary Note 1 for detailed risk of bias assessment.

**Table 2:** Critical appraisal of the seven selected prediction modelling studies based on the PROBAST checklist^25^

Study ID	Risk of bias				Applicability			Overall	
	Participant selection	Predictors	Outcome	Analysis	Participant selection	Predictors	Outcome	Risk of Bias	Applicability
Choi 2007	?	?	?	–	?	+	+	–	?
Hall 2001	–	?	–	?	–	+	–	–	–
Hirsch 2001/2004	–	+	–	–	–	+	–	–	–
Lim 2014	–	?	–	?	?	+	?	–	?
Metting 2016	+	+	–	–	–	+	+	–	–
Schneider 2015	+	+	?	–	+	+	+	–	+
Tomita 2013	+	?	–	–	–	+	+	–	–

(+) = low risk of bias or applicability concern, (?) = unclear risk of bias or applicability concern, (–) = high risk of bias or applicability concern

*PROBAST* Prediction model Risk Of Bias ASsessment Tool

### Model performance and validation

Three studies reported model performance using classification measures (Table 1),^15,19,21^ whilst three reported model discrimination using the area under the receiver operating characteristic curve (AUROC), which ranged from 0.61 to 0.82.^14,18,20^ None of the studies reported model calibration.

Hirsch et al.^17^ conducted internal validation, but did not report model performance. Metting et al.^19^ conducted an internal (10-fold cross) validation and external validation of the final decision tree using data from a different asthma/COPD referral service within the Netherlands. Model performance (derived from available data; no confidence intervals (CIs) available) was similar in the derivation (sensitivity 0.79, specificity 0.75) and validation datasets (sensitivity 0.78, specificity 0.60).^19^ Five studies reported no validation, with model performance likely to be over-estimated in these cases.^14,15,18,20,21^

### Model presentation

Of the six studies that derived a prediction model using logistic regression, four presented a scoring system,^14,17,18,21^ one a web-based clinical calculator^20^ and one presented model output from which a probability could be calculated.^15^ The decision tree had six ‘branches’ of predictors that led to a probability of asthma, though this approach limited the number of predictor combinations.^19^

### Model outcome measures

Four studies based their outcome measure on bronchial challenge testing;^14,18,20,21^ an asthma diagnosis was indicated by a 20% fall in forced expiratory volume in 1 s (FEV_1_) from baseline after stepwise inhalation of methacholine up to a maximum 8 mg/ml ^21^ or 16 mg/ml.^14,18,20^

Expert opinion informed the outcome in two studies.^17,19^ Hirsch et al.^17^ used a panel of three experts, whilst Metting et al.^19^ used one of ten respiratory specialists to make a diagnosis. In one study, healthcare providers made an asthma diagnosis when a child demonstrated reversible episodic symptoms, indicated by spirometry or symptom resolution.^15^

### Description of predictor variables

**Fig. 2:**
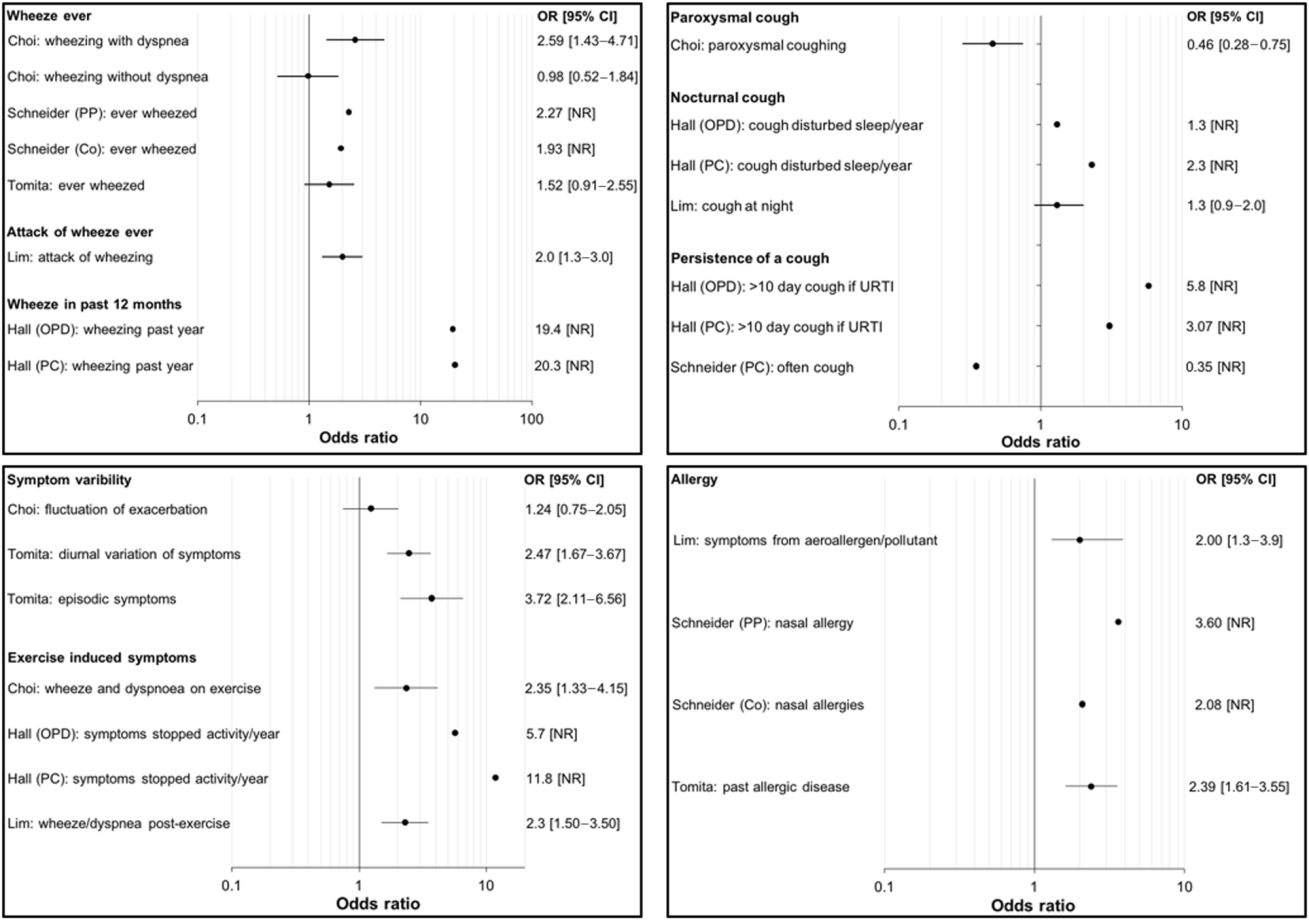
Forest plots demonstrating the strength of association of predictor variables against the outcome asthma. Not all studies had extractable data. PP = private practice, Co = combined dataset (private practice and primary care), OPD = out-patient department, PC = primary care. Confidence intervals were not reported for all estimates, indicated by [NR]. No overall estimates were produced as meta-analysis was not possible

The clinical prediction models combined between 4 (ref. ^15^) and 22 (ref. ^19^) predictors to estimate the probability of asthma. Three studies collected data from questionnaires only.^14,15,18^ The remaining studies collected a wider range of clinical data, though not all of the information was included in model development (Table 3). Figure 2 illustrates the strength of association between predictors included in the prediction models and the outcome, asthma. The most common predictors were wheeze, cough, symptom variability and allergy. Estimates for individual predictors were unavailable from two studies.^17,19^

**Table 3:** Predictors considered in each of the seven included prediction modelling studies

Predictors	Choi	Hall	Hirsch	Lim	Metting	Schneider	Tomita
Patient demographics							
Age	x	x	✓	x	✓	✓	x
Sex	x	x	✓	x	△	△	x
Weight/height/body mass index	x	–	–	x	△	–	x
Symptoms							
Wheeze	✓	✓	✓	✓	✓	✓	✓
Cough	✓	✓	–	–	–	✓	–
Night cough	–	✓	✓	✓	–	–	–
Breathlessness	✓	–	✓	–	△^a^	△	–
Respiratory infection	✓	–	–	✓	–	✓	–
Symptoms disrupting sleep	–	–	✓	–	△^a^	△	–
Symptom variability	✓	–	–	–	–	–	✓
Exercise-induced symptoms	✓	✓	–	✓	–	△	X^a^
Allergeninduced symptoms	–	–	–	✓	–	–	–
Medical history							
Smoking	x	–	✓	x	✓	△	x
History of allergy or atopy	–	x	✓	–	✓	✓	✓
Family history of allergy or atopy	–	x	–	–	△	–	✓
History of asthma/attack	–	x	✓	–	–	–	–
Asthma medication use	–	x	✓	–	△^a^	✓	–
Family history of asthma	–	x	✓	–	△	–	–
Findings from clinical examination and investigation							
Wheeze on auscultation	–	–	–	–	–	–	✓
Lung function	–	–	–	–	✓	–	x
Fractional exhaled nitric oxide	–	–	–	–	–	✓	x
Blood eosinophils	–	–	–	–	–	–	x
Serum IgE	–	–	–	–	–	–	x

Predictors are grouped to demonstrate commonalities between clinical prediction models. Each study constructed variables using different questions/measurements (Supplementary Table 3). (✓) = predictor included in final clinical prediction model. (△) = predictor not in final prediction model: excluded *during* modelling. (x) = predictor not in final prediction model: excluded *before* modelling. (–) = predictor was not measured/collected

^a^ Information was incorporated within a validated asthma questionnaire, but not analysed separately

Participant age was collected in all studies, but only considered in the model development of two studies.^17,20^ The decision tree used age of onset of respiratory symptoms in five of six branches.^19^ Male sex was associated with asthma in one model.^17^

Wheeze as a symptom was used in five clinical prediction models,^14,15,17–19^ though six different questions were used. Despite wide variation in how wheeze was recorded between studies, the magnitude of association between wheeze variables and asthma outcome were similar (Fig. 2) in four of five studies. The exception was Hall et al.^15^ whose reported estimates were much greater than other studies.

Cough was included in five of seven prediction models and asked about in three different ways (Fig. 2).^14,15,17,18,20^ Variables for cough were not clearly predictive for asthma in four studies.^14,17,18,20^ In contrast, Hall et al.^15^ reported that a cough lasting beyond 10 days after a cold was associated with asthma (odds ratio (OR) 5.8 (outpatients); OR 3.1 (primary care), CIs not reported), despite cough in children commonly taking over 10 days to settle.^24^

Respiratory tract infection was included in four prediction models, though was of unclear value as all studies were judged at high risk of bias.^14,17,18,20^

Being woken by chest tightness was associated with asthma in one study.^17^ Waking up because of cough in the past year was associated with asthma in one study at high risk of bias, though the lack of CIs makes the precision of estimates unclear.^15^ Symptoms disturbing sleep were not predictive in two other models.^19,20^

Episodic symptoms and diurnal variation were associated with asthma in one study,^21^ yet Choi et al.^14^ found ‘fluctuation of exacerbation and improvement’ was not associated with asthma (OR 1.24, 95% CI 0.75–2.05). Exercise-induced symptoms were associated with asthma in three studies.^14,15,18^ However, ‘dyspnoea on exertion’ was not significant in one study.^20^

The presence of allergy/atopic disease was predictive of asthma in five studies.^17–21^ Five of six decision tree branches included the presence/absence of allergy;^19^ past allergic disease, respiratory symptoms triggered by aeroallergens/pollutants and nasal allergy were significantly associated with asthma (Fig. 2).^18,20,21^

Current use of asthma medication was asked about and valuable in two studies,^17,20^ whilst past asthma attack was recorded by one study.^17^

Participants who smoked scored ‘−1’ in the prediction model by Hirsch et al.^17^ ‘never smoked’ and a ratio of FEV_1_ by forced vital capacity (FEV_1_/FVC) <70%, formed one of six decision tree branches leading to asthma.^19^ Four studies collected smoking data, but did not include it in their analysis.^14,18,20,21^

Family history of asthma was included by one study,^17^ but having a ‘close relative with allergic diseases’ was not associated with asthma in another (OR 1.19, 95% CI 0.73–1.93).^21^

Only Tomita et al.^21^ incorporated information from clinical examination. Wheeze heard on auscultation was associated with asthma (OR 3.68, 95% CI 1.78–7.62).^21^

FEV_1_/FVC was included in all branches that led to asthma in the decision tree.^19^ Bronchodilator reversibility was used in four out of six branches, though in contrast to guideline recommendations,^5–7^ two branches included reversibility of <7%.^19^ Schneider et al.^20^ included fractional exhaled nitric oxide (FeNO) as the main predictor in their clinical prediction models. Tomita et al^21^ collected relevant data but did not include in model development.

## Discussion

This systematic review identified seven clinical prediction models to support the diagnosis of asthma in primary care. All studies were judged to be at high risk of bias and cannot be recommended for diagnosing asthma in routine clinical practice. Wheeze, allergy, allergic rhinitis, symptom variability and exercise-induced symptoms were associated with asthma and should be considered as predictors in future prediction models. Cough, respiratory tract infection and nocturnal respiratory symptoms were inconsistently associated with asthma.

The use of Checklist for critical Appraisal and data extraction for systematic Reviews of prediction Modelling Studies (CHARMS) and Prediction model Risk Of Bias ASsessment Tool (PROBAST), systematic review frameworks specific for prediction models, in undertaking this review ensured each step was conducted to international standards. PROBAST was yet to be published, but we used it for risk of bias assessment as it was purposefully developed for reviews of prediction models by the Cochrane Prognosis Group, and had been successfully piloted.^25,26^ We reduced the possibility of reporting bias by duplicate, independent data extraction and risk of bias assessment. We planned to evaluate the overall quality of evidence using the Grading of Recommendations Assessment, Development and Evaluation (GRADE) system. Originally designed for reviews of intervention studies, GRADE has been adapted for reviews of prognostic studies, though not specifically for prediction models.^27,28^ Consequently, in its current form we did not find GRADE to be a suitable tool for our systematic review and decided not to use it. Future research should consider how to adapt GRADE so that it can be used for reviews of clinical prediction models.

We searched databases from 1 January 1990, having found no relevant literature before this date in preliminary searches. Our decision to search five databases was informed by the strategies of similar systematic reviews,^29,30^ but despite this we may have missed some relevant studies.

Restricting the population of interest to primary care (or equivalent) populations limited the number of studies we could include. Asthma may be diagnosed in both primary and secondary care, and current guidelines present diagnostic algorithms irrespective of clinical setting.^5–7^ However, the diagnostic value of symptoms, signs and tests vary depending on the setting in which they are used,^31^ and the general approach to making a diagnosis differs, as secondary care tend to see referred patients.^13^ As most diagnoses occur in non-specialist settings,^4^ we opted to focus on clinical prediction models derived from primary care participants. The degree to which study participants presented with undifferentiated symptoms was unclear in some studies. We sought additional information about the country of origin and made decisions based on team discussion to mitigate this uncertainty.

National and international guidelines are consistent in their advice to build up evidence to support a diagnosis of asthma based on history, examination, investigations and when necessary, a monitored trial of treatment.^5–7^ The Global Initiative for Asthma describes a characteristic pattern of symptoms (wheezing, shortness of breath, cough, chest tightness varying over time and in intensity) as indicative of asthma.^7^ Our included clinical prediction models endorse wheeze and symptom variability as potentially valuable predictors; however, cough and breathlessness were inconsistently associated with asthma. This inconsistency may in part have arisen from the different ways in which predictors were defined. For example, participants were asked about coughs that were variously ‘paroxysmal’, ‘nocturnal’, ‘daytime’, ‘often’ in the different studies limiting the comparison between prediction models and preventing meta-analysis. Additionally, patients and parents understand and describe symptoms differently from clinicians (and researchers),^32^ and future studies should choose reliable terms when phrasing questions about symptoms.^33^

Another reason for the inconsistent association between predictors and asthma observed in the included studies may be the imperfect nature of the outcome measure (reference standard) available for asthma. There is no universally accepted method to deal with an imperfect reference standard.^34^ Subsequently, in asthma diagnostic research it is not uncommon for different reference standards to be used between studies. For instance, in a systematic review reporting the accuracy of FeNO for asthma diagnosis, included studies were found to have substantial heterogeneity in the reference standards used.^35^ In this review, four studies used methacholine bronchial provocation, considered to be the best available reference standard for asthma, though it is known to be better at ruling out, rather than ruling in the diagnosis.^36^ The remaining studies used clinician judgement to classify those with/without asthma, a valid solution in the face of an imperfect reference standard,^34^ but highly dependent on the performance, consistency and agreement of the clinicians. Understanding that the performance of a prediction model for asthma diagnosis depends so heavily on the outcome measure chosen, future studies should consider recommendations to move away from the umbrella term ‘asthma’, instead focussing on identifying ‘treatable traits’ as failure to recognise asthma as an aggregate diagnosis is likely to limit any improvement in diagnostic accuracy gained from a clinical prediction model.^4,37^

This review highlights the paucity of current evidence to inform diagnostic algorithms. A validated clinical prediction model for asthma diagnosis could help healthcare professionals improve the accuracy of a diagnosis by guiding decision-making and reducing variability between clinicians. That only two studies considered diagnostic tests as candidate predictors was disappointing given the potential for prediction models to combine information from a clinical history, physical examination and tests. Failure to confirm the presence of asthma with objective tests has been implicated in the widespread misdiagnosis.^1^ So, on a practical level, a validated prediction model that guides a clinician in the questions to ask, and the test(s) required to confirm or refute an asthma diagnosis, is likely to be most useful.

Future attempts at model derivation for asthma diagnosis should be informed by recognised standards such as the Transparent Reporting of a multivariable prediction model for Individual Prognosis Or Diagnosis (TRIPOD).^38^ Prediction models should undergo internal and external validation and report model performance using calibration and discrimination measures.^39^ In this review, none of the included studies reported model calibration. Model validation was completed by only two studies, a finding that matches the wider literature.^40^ Finally, strategies to implement the validated model in routine clinical practice need to be developed, piloted and evaluated,^41^ to assess impact on clinical outcomes.^40^

In conclusion, existing clinical prediction models to support clinicians in making a diagnosis of asthma in primary care are at high risk of bias and thus of limited clinical value. Wheeze, symptom variability and the presence of other allergic disease were associated with asthma diagnosis. Informed by this review, future studies should address the limitations identified and follow established methods to derive and validate a prediction model of value to clinicians. Establishing a data-driven approach to asthma diagnosis could resolve current discrepancies in guidelines and enable the unacceptable level of asthma misdiagnosis to be reduced.

## Methods

The systematic review was registered with PROSPERO (CRD42018078418). Detailed methods were described in the published protocol,^42^ with salient points presented here. We followed the CHARMS^39^ and Preferred Reporting Items for Systematic Review and Meta-Analysis (PRISMA).^43^

### Study eligibility criteria

#### Population

Children or adults presenting with symptoms suggestive of asthma in primary care.

#### Intervention

Any clinical prediction model designed to aid the diagnostic decision-making of a healthcare professional during the assessment of an individual with symptoms suggestive of asthma.

#### Comparator

Not applicable.

#### Outcome

The primary outcome to be predicted was the probability of an asthma diagnosis. We included studies that presented a prediction model, or equivalent statistical method, that allowed the probability of asthma to be calculated for an individual. To be included, the study had to use an outcome based on an internationally recognised definition for asthma (as available, for instance, from the Global Initiative for Asthma.^7^)

#### Timing

Any diagnostic prediction model that provides an estimate for the probability that asthma is present at the time of clinical assessment.

#### Setting

We included any clinical prediction model designed for use in a primary care population or equivalent (defined as any setting where undifferentiated health problems are presented to healthcare professionals).^13^

#### Study type

We included prediction model derivation studies (with or without external validation) and external model validation studies.^39^ Randomised controlled trials, cohort studies (prospective or retrospective), cross-sectional, nested case–control and case–cohort studies were eligible for inclusion.^39^

### Exclusion criteria

Studies were excluded if:

1. Variables were not combined to produce a diagnostic estimate
2. Publication occurred before 1 January 1990 (preliminary searches identified no relevant citations before this date)
3. Variables used in the clinical prediction model were not clearly reported, or unavailable in routine clinical practice (for example genetic tests)
4. Separate outcomes for asthma were not reported or the asthma outcome was not extractable
5. The prediction model was derived to predict the future risk of asthma
6. Over half of study participants were children <5 years old (because of the overlap between asthma and viral associated wheeze in this age group)
7. Non-original studies such as editorials, expert views.

### Information sources and search strategy

We searched Medline, Embase, CINAHL, TRIP (https://www.tripdatabase.com) and US National Guidelines Clearinghouse (https://www.guideline.gov) databases from 1 January 1990 to 23 November 2017. The search strategy (Supplementary Table 2) combined published searches for prediction models^44,45^ with Cochrane Airways asthma search terms.^46^ Forward and backward citation searching was completed. No language restrictions were used. Studies were translated when necessary.

### Study selection

Retrieved records were de-duplicated, screened and managed using Covidence (https://www.covidence.org). Two reviewers (L.D., A.B.) independently screened titles and abstracts. Full-text copies of all relevant records were obtained. Two reviewers (L.D., S.McL.) independently assessed each full-text record for eligibility. Discrepancies were arbitrated by discussion (H.P., S.L. and A.S.).

### Data collection process

A standardised data extraction form was developed using CHARMS and piloted.^39^ Two reviewers (L.D., S.McL.) independently extracted data from included studies, with disagreements resolved by third reviewer (H.P., S.L. or A.S.). Study authors were contacted if further information or clarification was required. Data were summarised in descriptive tables (Supplementary Table 3).

### Critical appraisal of individual studies

Two reviewers (L.D., S.McL.) used the PROBAST to independently evaluate risk of bias and concerns about applicability before reaching a consensus for each included study.^25^ According to PROBAST, risk of bias assessment is guided by 20 signalling questions across four domains; participant selection, predictors, outcome and analysis. Each domain is scored low, high or unclear risk of bias and combined to provide an assessment for each study. If a study scores high risk of bias for any domain, PROBAST advises the study to be rated high risk of bias overall. The extent to which each study matched the review question was assessed using PROBAST applicability concern questions. Three domains were assessed; participant selection, predictors and outcome, leading to an overall rating for applicability.

### Data synthesis and summary measures

Results were summarised by narrative synthesis as between-study heterogeneity precluded meta-analyses. We summarised the final model presentation and available measures of overall performance, including calibration, discrimination and classification parameters, from each included study. We appraised the strength of association of predictors used in each model against the outcome (asthma) by comparing regression coefficients and odds ratios.

### Evaluating confidence in cumulative evidence

We planned to report the overall quality of evidence using GRADE. However, in a change from our protocol, we decided to omit the use of GRADE, as without an adaptation for prediction modelling studies, we did not find it to be a suitable tool.^27^ Assessment of publication bias was not completed due to heterogeneity between studies.

## Supplementary Information

Supplementary Material

## Data Availability

Any data generated or analysed are included in this article and the Supplementary files. Additional data may be available from the corresponding author on reasonable request.

## Supplementary information

**Supplementary information** accompanies the paper on the *npj Primary Care Respiratory Medicine* website (10.1038/s41533-019-0132-z).
