# Supplementary material for: Systematic review of clinical prediction models to support the diagnosis of asthma in primary care
Source: NPJ Prim Care Respir Med. 2019 May 9;29:19. doi: 10.1038/s41533-019-0132-z (PMC6509212; doi:10.1038/s41533-019-0132-z)
Supplement: Supplementary file 1 — Supplementary Material [file 41533_2019_132_MOESM1_ESM.pdf]

Supplementary Note 1: Critical appraisal of included studies

Critical appraisal was conducted using PROBAST[1], which includes an assessment of the risk of bias and applicability of each study.

Risk of Bias

All the included studies were judged to be at high risk of bias. Bias was introduced by various means, though certain limitations were shared by several studies.

Participant selection

Participant selection was at low risk of bias for three studies.[2,3,4] Choi et al[5] gave little detail about inclusion criteria and was rated unclear. Three studies were judged high risk of bias; Hall et al[6] included a high proportion (40%) of children aged between 6 months and 5 years, a group notoriously difficult to make a diagnosis of asthma. Hirsch et al[7] conducted analyses on a small, selected sample drawn from a cross-sectional survey of the general population. Lim et al[8] also used an overly selected sample, by only including adults who had an FEV_1 greater than 70% of their predictive value on spirometry.

Predictors

Three studies were rated at low risk of bias for the selection and measurement of predictors used in their models.[2,3,7] The remaining four models had unclear risk of bias as there was insufficient information to judge if predictor assessments had been made without knowledge of outcome data.[4-6,8]

Outcome

Five studies were rated at high risk of bias for the outcome used.[2,4,6-8] In two studies the outcome was assessed without being blind to predictors; Lim et al[8] stated that responses to the questionnaire used as their predictor variables were considered when deciding the outcome. Metting et al[2] used spirometry measures to inform both predictor variables and the outcome.

The timing of assessments introduced bias in one study. Tomita et al[4] started inhaled corticosteroid treatment after the first assessment of participants (when the predictor variables were measured) which is likely to have influenced the results of their outcome measure, methacholine bronchial provocation, performed up to eight weeks later.

The outcome measure used by Hall et al[6] had several potential sources of bias. Their outcome was based on the judgement of healthcare providers at each recruitment site, yet it was unclear how many providers were used, their medical background or if any training was provided. Providers based their decision on reversible airflow obstruction, measured in two ways; either clinically by the relatively subjective resolution of symptoms, or objectively by spirometry. Yet, spirometry is difficult to achieve in young children,[9] and in this study, was only attempted in children aged seven or above. Therefore, data for spirometry was only available for 80 of the 211 participants.

Hirsch et al[7] combined the assessment of three experts to assign a probability of asthma for each of the 180 participants based on a clinical assessment which included reversibility and bronchial challenge tests. Rather than base their outcome on the result of an objective test, participants were categorised with asthma if the consensus probability of asthma was 50% or greater, introducing bias into their measure.

Two studies were at unclear risk of bias for outcome based on a lack of information regarding the timing of predictor and outcome measurement.[3,5] In addition, Choi et al[5] did not report if the outcome measure was assessed blind to the predictor variables and the same outcome was not used for every participant as asthma could be classified based on reversibility or bronchial provocation.

Analysis

Five studies were rated at high risk of bias due to the methods used in analysis.[2-5,7] Seven participants were excluded from the model building of Schneider et al[3] with a further 81 missing from the analysis in the combined model. In addition, selection of candidate

predictors in the model was based on univariate analysis, which is known to introduce bias when completing multivariable modelling.[10] Similarly, Tomita et al[4] initially used univariate analysis in selecting their candidate predictors and did not include all the enrolled participants in their analysis. Choi et al[5] also did not explain their handling of missing data, despite 56 participants not having data for six questions. Additionally, the score (weights) attached to each predictor in their model did not match the regression output.[5] Hirsch et al[7] excluded 21 participants from their derivation sample due to missing data, leaving 180 participants and only 84 with the outcome. The events per variable was small (5.6), and at high risk of bias. Metting et al[2] categorised continuous variables and excluded 135 individuals who had data missing at random.

Two studies were rated at unclear risk of bias for analysis, as information regarding the handling of missing data, selection of predictor variables, model overfitting and the weights assigned to each predictor was not reported.[6,8]

Applicability

Overall, one study had low overall applicability concern,[3] four had high[2,4,6,7] and two were rated unclear.[5,8] The selection of participants was the major reason for studies not closely matching the review question.

Participant selection

High concern for the participants or selection method not matching the review question was found in four studies.[2,4,6,7] Hirsch et al[7] conducted a postal survey, inviting all adults to take part, therefore the initial sample was not confined to those with symptoms suggesting asthma. Hall et al[6] included children below the age of five years, which made up 40% of the primary and secondary care samples they used. Metting et al[2] included participants who had been referred by their GP to receive further assessment, although the asthma/COPD referral service is situated at the interface between primary and secondary care. 39% of the 4129

participants recruited by Tomita et al[4] had abnormalities on x-ray, indicating that a large number of participants presented with symptoms suggesting an alternative diagnosis to asthma.

Two studies at unclear applicability concern, recruited from hospital outpatient settings in South Korea.[5,8] Each sample was judged to be equivalent to primary care, given the limited availability of primary care services in the country.[11] However, it is likely that patient characteristics would be different from those presenting to primary care in a country where it is established.

Predictors

The definition or assessment of the predictors matched the review question closely in all included studies.

Outcome

The outcome in four studies closely matched the systematic review question and were rated low applicability concern.[2-5] The applicability of one study was unclear due to the timing of the outcome assessment in relation to initial testing being unreported.[8] Two studies were at high applicability concern; the inclusion of children below the age of five years by Hall et al[6] meant that making an accurate diagnosis of asthma for all participants was unlikely; Hirsch et al[7] categorised individuals as having asthma if their probability was 50% or above, which did not closely match the review question.

References

[1] Moons, K.G.M, et al. PROBAST: a tool to assess risk of bias and applicability of prediction model studies: explanation and elaboration. Ann Intern Med 170 (1) W1-33 (2019) doi: 10.7326/M18-1377

[2] Metting, E.I. *et al*. Development of a diagnostic decision tree for obstructive pulmonary diseases based on real-life data. *ERJ Open Res*. 2(1), 00077-2015 (2016)

[3] Schneider, A. Wagenpfeil, G. Jörres, R.A. & Wagenpfeil, S. Influence of the practice setting on diagnostic prediction rules using FENO measurement in combination with clinical signs and symptoms of asthma. *BMJ open*. 5(11), e009676 (2015)

[4] Tomita, K. *et al*. A scoring algorithm for predicting the presence of adult asthma: a prospective derivation study. *NPJ Prim Care Respir Med*. 22(1), 51 (2013)

[5] Choi, B.W. *et al*. Easy diagnosis of asthma: computer-assisted, symptom-based diagnosis. *J Korean Med Sci*. 22(5), 832-8 (2007)

[6] Hall, C.B. Wakefield, D. Rowe, T.M. Carlisle, P.S. & Cloutier, M.M. Diagnosing pediatric asthma: validating the Easy Breathing Survey. *J Pediatr*. 139(2), 267-72 (2001)

[7] Hirsch, S. Frank, T.L. Shapiro, J.L. Hazell, M.L. & Frank, P.I. Development of a questionnaire weighted scoring system to target diagnostic examinations for asthma in adults: a modelling study. *BMC Fam Pract*. 5(1), 30 (2004)

[8] Lim, S.Y. Jo, Y.J. & Chun, E.M. The correlation between the bronchial hyperresponsiveness to methacholine and asthma like symptoms by GINA questionnaires for the diagnosis of asthma. *BMC Pulm Med*. 14(1), 161 (2014)

[9] Ducharme, F.M. Sze, M.T. & Chauhan, B. Diagnosis, management, and prognosis of preschool wheeze. *Lancet*. 383(9928), 1593-604 (2014)

[10] Sun, G.W. Shook, T.L. & Kay, G.L. Inappropriate use of bivariable analysis to screen risk factors for use in multivariable analysis. *J Clin Epidemiol*. 49(8), 907-16 (1996)

[11] Kwon, S. Lee, T-J. & Kimm, C-Y. Republic of Korea health system review. Health Systems in Transition. 5(4) (2015)

Supplementary Table 1: Studies excluded after full text screening

Study	Reason for exclusion
Bansal 2001	Present a method of identifying asthma patients from a sample of patients prior to entering a trial, not for use in clinical consultation.
Barnes 1999	Validates algorithm of Panhuysen. Use in identifying asthma patients in an epidemiological study. Not for use in clinical consultation
Bicherakhov 1994	Outcomes for asthma are not separate or data relating to the asthma outcome is not extractable.
Bonner 2006	Case detection in a pre-school sample, not for use in clinical consultation.
Burge 1999	Investigate the interpretation of peak expiratory flow measurements using neural network; the predictive value of more than one variable was evaluated but not combined to produce a diagnostic estimate.
Carroll 2012	The predictive value of more than one variable was evaluated but not combined to produce a diagnostic estimate
Cave 2016	Algorithms for identifying patients in electronic health record, not for use in clinical consultation.
Deng 2010	The focus is on case identification in population based studies, not for use in clinical consultation.
Eysink 2005	The CPM was derived to predict future risk of asthma and over half of the participants included were children less than five years old
Fukuhara 2011	Patients included were not from primary care or equivalent setting. The reference standard used is not based on an internationally recognised definition of asthma.
Grassi 2003	Population based screening questionnaire, not a CPM.
Holleman 1993	Patients included were not from primary care or equivalent setting. Outcomes for asthma are not separate or data relating to the asthma outcome is not extractable.
Jamrozik 2009	The CPM was derived to predict future risk of asthma
Jones 2004	School based case detection, not for use in clinical consultation.
Kable 2001	Variables included in the model are not clearly reported (doesn't allow the probability of asthma to be calculated for other individuals)
Lee 2015	Patients included were not from primary care or equivalent setting.
Li 1998	The algorithm was created based on expert opinion, not a CPM.
Liebhart 1998	Patients included were not from primary care or equivalent setting.
Ma 2017	Outcomes for asthma are not separate or data relating to the asthma outcome is not extractable.
Menezes 2015	Does not present a CPM that could be used in clinical practice and the reference standard used is not based on an internationally recognised definition of asthma.
Murray 2017	Not a CPM - the original algorithm tested in this study was derived by economic modelling with expert recommendation
Panhuysen 1998	Algorithm designed for identifying those with asthma in a sample recruited for a clinical trial, not for use in clinical consultation.

Pralong 2013	Patients included were not from primary care or equivalent setting.
Redline 2003	Develop a score to screen school children (with and without symptoms) likely to have asthma so that they can be referred for a diagnostic assessment, not for use in clinical consultation.
Redline 2004	Develop a score to screen school children (with and without symptoms) likely to have asthma so that they can be referred for a diagnostic assessment, not for use in clinical consultation.
Remes 2002	Variables used in the model are not clearly reported
Rother 2015	Patients included were not from primary care or equivalent setting.
Schneider 2003	The algorithm was created based on expert opinion, not a CPM.
Schneider 2012	Does not present a CPM that could be used in clinical practice.
Sistek 2001	The reference standard used is not based on an internationally recognised definition of asthma.
Sunyer 2007	Does not present a CPM that could be used in clinical practice.
Thiadens 1998	Data relating to the asthma outcome is not extractable.
Thiadens 2000	The population analysed is not representative of a primary care population
Thorat 2017	Patients included were not from primary care or equivalent setting.
Topalovic 2017	Investigate algorithms for lung function test interpretation.
Torchio 2005	Patients included were not from primary care or equivalent setting and data relating to the asthma outcome is not extractable.
Tyagi 2014	Does not present a CPM that could be used in clinical practice.
Vandenplas 2005	Patients included were not from primary care or equivalent setting.
Wahn 2000	Non-original study – review article
Wahn 2004	Non-original study – review article
Wever-Hess 1999	Participants were children aged 0-4 years.
Xi 2015	Describe algorithms for searching electronic databases not for clinical use.
Yu 2004	The reference standard used is not based on an internationally recognised definition of asthma.
Zolnoori 2012a	Outcomes for asthma are not separate or data relating to the asthma outcome is not extractable and variables used in the model are not available in routine clinical practice.
Zolnoori 2012b	Outcomes for asthma are not separate or data relating to the asthma outcome is not extractable and variables used in the model are not available in routine clinical practice.

References:

Barnes, K. C. *et al.* Physician-derived asthma diagnoses made on the basis of questionnaire data are in good agreement with based diagnoses and are not affected by objective tests. Journal of Allergy and Clinical Immunology. 104(4 I), 791-796 (1999)

Bansal, A. *et al.* A simple diagnostic index for asthma. Clinical & Experimental Allergy. 31(5), 756-60 (2001)

Bicherakhov, S.K. Plavskii, L.V. & Shalagin, A.I. An algorithm approach to the diagnosis of the forms of bronchial asthma (based on anamnestic data). [Russian] Likars'ka sprava / Ministerstvo okhorony zdorov'ia Ukrainy. (1), 89-91 (1994)

Bonner, S. *et al*. Validating an asthma case detection instrument in a Head Start sample. Journal of School Health. 76(9), 471-8 (2006)

Burge, P.S. *et al*. Development of an expert system for the interpretation of serial peak expiratory flow measurements in the diagnosis of occupational asthma. Midlands Thoracic Society Research Group. Occupational & Environmental Medicine. 56(11), 758-64 (1999)

Carroll, A.E. *et al*. Increased physician diagnosis of asthma with the child health improvement through computer automation decision support system Pediatric, Allergy, Immunology, and Pulmonology. 25 (3), 168-171 (2012)

Cave, A.J. *et al*. Development of a validated algorithm for the diagnosis of paediatric asthma in electronic medical records npj Primary Care Respiratory Medicine. 26 (2016)

Deng, X. *et al.* Development of a nomogram for identification of asthma among adults in epidemiologic studies. Annals of Allergy, Asthma, & Immunology. 105(3), 203-210 (2010)

Eysink, P. E. *et al.* Accuracy of specific IgE in the prediction of asthma: development of a scoring formula for general practice. British Journal of General Practice. 55(511), 125-131 (2005)

Fukuhara, A. *et al.* Validation study of asthma screening criteria based on subjective symptoms and fractional exhaled nitric oxide. Annals of Allergy, Asthma and Immunology. 107 (6), 480-486 (2011)

Grassi, M. Rezzani, C. Biino, G. & Marinoni, A. Asthma-like symptoms assessment through ECRHS screening questionnaire scoring. Journal of clinical epidemiology. 56(3), 238-247 (2003)

Holleman, D.R., Jr. Simel D.L. & Goldberg, J.S. Diagnosis of obstructive airways disease from the clinical examination. Journal of General Internal Medicine. 8(2):63-8 (1993)

Jamrozik, E. Knuiman, M.W. James, A. Divitini, M. & Musk, A.W. Risk factors for adult-onset asthma: A 14-year longitudinal study. Respirology. 14 (6), 814-821 (2009)

Jones, C.A. *et al.* A school-based case identification process for identifying inner city children with asthma: The Breathmobile Program. Chest.125, 924-34 (2004)

Kable, S. *et al.* Childhood asthma: can computers aid detection in general practice? Br J Gen Pract, 51(463), 112-116 (2001)

Lee, Y.S. New scoring system for the differentiation of chronic obstructive pulmonary disease and asthma. Respirology. 20(4), 626-32 (2015)

Li, J. T. *et al*. Algorithm for the diagnosis and management of asthma: a practice parameter update. Annals of Allergy, Asthma, & Immunology. 81(5), 415-20 (1998)

Liebhart, J. & Dor, A. Diagnostic standard for differentiation between bronchial asthma and chronic obstructive pulmonary disease. [Polish] Pneumonologia i alergologia polska : organ Polskiego Towarzystwa Ftyzjopneumonologicznego, Polskiego Towarzystwa Alergologicznego, i Instytutu Gruzlicy i Chorob Pluc. 66(7-8),373-382 (1998)

Ma, T.T. *et al*. Predictive value of respiratory symptoms for the diagnosis of pollen-induced seasonal asthma among children and adults in Inner Mongolia. Therapeutics and Clinical Risk Management. 13, 967-974 (2017)

Menezes, J. & Gusmao, C. Development of a Mobile System Decision-support for Medical Diagnosis of Asthma in Primary Healthcare—InteliMED. Studies in health technology and informatics. 216, 959 (2015)

Murray, C. *et al.* Diagnosis of asthma in symptomatic children based on measures of lung function: an analysis of data from a population-based birth cohort study. The Lancet Child and Adolescent Health. 1 (2), 114-123 (2017)

Panhuysen, C.I. Bleecker, E.R. Koeter,G.H. Meyers, D.A. & Postma, D.S. Characterization of obstructive airway disease in family members of probands with asthma. An algorithm for the diagnosis of asthma. American Journal of Respiratory & Critical Care Medicine. 157(6 Pt 1):1734-42 (1998)

Pralong, J.A. *et al*. Screening for occupational asthma by using a self-administered questionnaire in a clinical setting. Journal of Occupational & Environmental Medicine. 55(5), 527-31 (2013)

Remes, S.T. Pekkanen, J. Remes, K. Salonen, R.O. & Korppi, M. In search of childhood asthma: questionnaire, tests of bronchial hyperresponsiveness, and clinical evaluation. Thorax. 57(2), 120-126 (2002)

Redline, S. Larkin, E. K. Kercsmar, C. Berger, M. & Siminoff, L.A. Development and validation of school-based asthma and allergy screening instruments for parents and students Annals of Allergy, Asthma and Immunology. 90(5), 516-528 2003.

Redline, S. *et al.* Development and validation of school-based asthma and allergy screening questionnaires in a 4-city study. Annals of Allergy, Asthma & Immunology, 93(1), 36-48 (2004)

Rother, A.K. *et al*. Diagnostic support for selected paediatric pulmonary diseases using answer-pattern recognition in questionnaires based on combined data mining applications-a monocentric observational pilot study PLoS ONE. 10 (8) (2015)

Schneider, A. Borst, M.M. Gerlach, F.M. & Szecsenyi, J. Suspected Diagnosis of Bronchial Asthma - Development of an algorithm for the diagnosis of asthma in general practice. [German] Zeitschrift fur Arztliche Fortbildung und Qualitatssicherung. 97 (7), 485-493 (2003)

Schneider, A.; Ay, M.; Faderl, B.; Linde, K.; Wagenpfeil, S. Diagnostic accuracy of clinical symptoms in obstructive airway diseases varied within different health care sectors Journal of Clinical Epidemiology. 65 (8), 846-854 (2012)

Sistek, D. *et al.* Clinical diagnosis of current asthma: predictive value of respiratory symptoms in the SAPALDIA study. *ERJ*, 17(2), 214-219 (2001)

Sunyer, J. *et al*. Asthma score: predictive ability and risk factors Allergy. 62(2),142-8 (2007)

Thiadens, H.A. *et al*. Identifying asthma and chronic obstructive pulmonary disease in patients with persistent cough presenting to general practitioners: descriptive study. *Bmj*, *316*(7140), 1286-1290 (1998)

Thiadens, H.A. *et al*. Asthma in adult patients presenting with symptoms of acute bronchitis in general practice. Scandinavian journal of primary health care, 18(3), 188-192 (2000)

Thorat, Y.T. Salvi, S.S. & Kodgule, R.R. Peak flow meter with a questionnaire and mini-spirometer to help detect asthma and COPD in real-life clinical practice: a cross-sectional study. npj Primary Care Respiratory Medicine, 27(1), 32 (2017)

Topalovic, M. *et al.* Automated Interpretation of Pulmonary Function Tests in Adults with Respiratory Complaints. Respiration. 93 (3), 170-178 (2017)

Torchio, M. *et al*. Validation of a knowledge-based expert system for the diagnostic assessment of dyspnoea. Minerva Pneumologica. 44 (1), 17-27 (2005)

Tyagi, A. & Singh, P. Asthma diagnosis and level of control using decision tree and fuzzy system. International Journal of Biomedical Engineering and Technology. 16(2), 169-181 (2014)

Vandenplas, O. *et al.* What are the questionnaire items most useful in identifying subjects with occupational asthma? European Respiratory Journal. 26(6):1056-63 (2005)

Wahn, U. Chuchalin, A. & Kowalski, M.L. Predictive and Early Diagnosis. *Allergy*, *55*(11), 1078-1080 (2000)

Wahn, U. Chuchalin, A. & Kowalski, M.L. Prediction and early diagnosis. In *Prevention of Allergy and Allergic Asthma*. 84, 128-134 (2004)

Wever-Hess, J. Kouwenberg, J.M. Duiverman, E.J. Hermans, J. & Wever, A.M. Prognostic characteristics of asthma diagnosis in early childhood in clinical practice Acta Paediatrica 88(8), 827-34 (1999)

Xi, N. *et al*. Identifying patients with asthma in primary care electronic medical record systems Chart analysis-based electronic algorithm validation study Canadian Family Physician. 61(10) (2015)

Yu, I.T. Wong, T.W. & Li, W. Using child reported respiratory symptoms to diagnose asthma in the community. Archives of Disease in Childhood. 89, 544-8 (2004)

Zolnoori, M. Fazel Zarandi, M.H. Moin, M. Heidarnezhad, H. & Kazemnejad, A. Computer-aided intelligent system for diagnosing pediatric asthma. Journal of Medical Systems. 36(2), 809-22 (2012)

Zolnoori, M. Zarandi, M.H. & Moin, M. Application of intelligent systems in asthma disease: designing a fuzzy rule-based system for evaluating level of asthma exacerbation Journal of Medical Systems. 36(4), 2071-83 (2012)

Supplementary Table 2: Search strategy for Medline

1	exp Asthma/
2	asthma$.mp.
3	(antiasthma$ or anti-asthma$).mp.
4	Respiratory Sounds/
5	wheez$.mp.
6	Bronchial Spasm/
7	bronchospas$.mp.
8	(bronch$ adj3 spasm$).mp.
9	bronchoconstrict$.mp.
10	exp Bronchoconstriction/
11	(bronch$ adj3 constrict$).mp.
12	Bronchial Hyperreactivity/
13	Respiratory Hypersensitivity/
14	((bronchial$ or respiratory or airway$ or lung$) adj3 (hypersensitiv$ or hyperreactiv$ or allerg$ or insufficiency)).mp.
15	OR/1-14
16	(Validat$ OR Predict$.ti. OR Rule$)
17	(Predict$ AND (Outcome$ OR Risk$ OR Model$))
18	((History OR Variable$ OR Criteria OR Scor$ OR Characteristic$ OR Finding$ OR Factor$) AND (Predict$ OR Model$ OR Decision$ OR Identif$ OR Prognos$))
19	(Decision$ AND (Model$ OR Clinical$ OR Logistic Models/))
20	(Prognostic AND (History OR Variable$ OR Criteria OR Scor$ OR Characteristic$ OR Finding$ OR Factor$ OR Model$))
21	Stratification.mp
22	ROC curve/
23	Discrimination.mp
24	Discriminate.mp
25	c-statistic.mp
26	c statistic.mp
27	Area under the curve.mp
28	AUC.mp OR Area Under Curve/
29	Calibration.mp OR Calibration/
30	Indices.mp
31	Algorithm.mp OR Algorithms/
32	Multivariable
33	OR/16-32
34	Asthma/di
35	Exp *Diagnosis/
36	(diagnos?s or diagnostic).tw.
37	OR/35-36
38	pre-school$.ti.
39	preschool$.ti.
40	infant$.ti.
41	newborn$.ti.
42	OR/38-41
43	15 and 33 and 37
44	43 not 42
45	Animals/ not Humans/
46	44 not 45
47	Editorial/
48	Letter/
49	47 or 48
50	46 not 49

Supplementary Table 3: Detailed information from included studies

Study
Choi 2007

Study population
Design: Cohort study (not clear if retro-/pro- spective)

Recruitment:
Method: Adult patients visited the hospital for "various respiratory symptoms such as dyspnoea, cough or wheezing"

Setting: Out-patient department of 6 hospitals

Location: South Korea (exact hospitals not disclosed)

Eligibility:
Inclusion: Adult (age range not reported) with "various respiratory symptoms"

Exclusion: Not reported

Study Dates: Not reported

Model development

Outcome
Definition: Asthma / Non-asthma

Measured:
Using spirometry, forced expiratory volume in one second (FEV1) was measured.

Patients with FEV1 of more than 70% of predictive value underwent a methacholine bronchial provocation test (MBPT), while the rest were evaluated for BDR to short-acting 2-agonist.

Type: Single endpoint

Same for all participants: No

Blind to predictors: Not reported

Timing: Not reported

Predictors
Description:
Participant asked 11 questions by a physician:

1. Have you had wheezing associated with dyspnea?

If yes to 1: Provoking factors:
- 1-1. Nocturnal aggravation
- 1-2. Cold air
- 1-3. Exercise
- 1-4. Upper respiratory infection
- 1-5. Smoke or air pollution
- 1-6. Concurrently with coughing

2. Have you had paroxysmal coughing?

3. Have you had dyspnea without wheezing?

4. Have you had wheezing without dyspnea?

5. Have you had fluctuation of exacerbation and improvement?

Assessed blind to outcome:
Not reported

Handling in the model:
All responses were binary variables

Sample
Participants: 302

Events per variable:
210 "asthma" 210 / 11 = 19.1
98 "non asthma" 98 / 11 = 8.9

Missing data: No missing data is reported.

Participants with any missing data:
Only those who answered 'yes' to question 1 were asked questions 1-1. to 1-6. (n = 246)
Therefore 56 had missing data for 6 of the variables used.

Methods for handling missing data:
Not reported

Model performance and Evaluation
Final model presentation:

1. wheezing with dyspnea?	2
If yes to 1, provoking factors	
1-1 Nocturnal aggravation	1
1-2. Cold air	1
1-3. Exercise	1
1-4 Upper resp infection	1
1-5. Smoke or air pollution	1
1-6. Concurrent coughing	1
2. Paroxysmal coughing?	1
3. Dyspnea w/o wheezing?	1
4. Wheezing w/o dyspnea?	1
5. Fluctuation of exacerbation/improvement?	2

Performance measures:
ROC analysis of total symptom scores AUC = 0.647±0.033

Performance of various cut-off values of total symptom score:

Cut-off	Sens	Spec
≥3	0.924	0.033
≥4	0.852	0.250
≥5	0.743	0.478
≥6	0.595	0.663
≥7	0.400	0.837
≥8	0.214	0.891
≥9	0.143	0.957
≥10	0.086	0.967
≥11	0.043	0.989

Focus on cut-off of ≥4. 12 combinations of variables can score ≥4. Range of sensitivity 13.8% and 56.2%; specificity was between 69.6% and 93.5%.

General information
COI:
Not reported.

Strengths:
Implementation of CPM into clinical practice was prioritised.

Limitations:
No validation.

Generalisability:
South Korean outpatient setting.

Model building
Modelling method:
Multivariate logistic regression

Model assumptions met:
Not reported

Selection of predictors:
Before modelling: Not reported
During modelling: Not reported

Shrinkage of predictor weights:
Not clear

Model Evaluation
Internal validation:
No internal validation reported

Method used: N/A
Number in validation sample: N/A
Number with outcome: N/A

External validation:
No external validation reported

If externally validated, was the model adjusted? N/A

Study	Study population	Model development			Model performance and Evaluation	General information
		Outcome	Predictors	Sample		
**Hall 2001**	**Design:**<br>Prospective cohort<br><br>Validated their *questionnaire* “easy breathing survey” in secondary care clinics.<br><br>The validated survey was then used in primary care clinics to assess the “validity of the survey for the diagnosis of asthma”<br><br>**Recruitment:**<br>*Method:*<br><br>‘Validation’ sites: Consecutive recruitment of participants from four secondary care outpatient clinics:<br>1. Pulmonology<br>2. Cardiology<br>3. Endocrinology<br>4. General Paediatrics<br><br>Primary care sites: Children who presented for any reason at six primary care sites.<br><br>*Location:* Hartford, Connecticut<br><br>**Eligibility:**<br>*Inclusion:* Children aged 6 months to 18 years.<br><br>*Exclusion:*<br>‘Validation sites’: Only patients new to the clinic, before they had been seen by pulmonology specialist.<br><br>‘primary care sites’: not stated<br><br>**Study Dates:**<br>June 1998 - 21 May 1999	**Definition:**<br>Asthma / No asthma<br><br>**Measured:**<br>“Considered when a child experienced episodic, recurrent (>2 episodes) airflow obstruction that was partially reversible either clinically (symptom resolution) or, if feasible, by spirometry and when other diagnoses had been excluded.”<br><br>**Type:** Combined<br><br>**Same for all participants:** No. Not all children were able to perform lung function tests.<br><br>**Blind to predictors:** Not reported<br><br>**Timing:** Not reported	**Description:**<br>Participant responses to four questions from the easy breathing survey.<br><br>1. Has your child had wheezing or whistling in the chest at any time in the last 12months? ("Wheeze")<br><br>2. Has your child awakened at night because of coughing in the last 12months? ("Nocturnal cough")<br><br>3. Has your child had coughing, wheezing or shortness of breath with exercise or activity and had to stop because of these symptoms at any time in the last 12 months? ("Exercise symptoms")<br><br>4. When your child has a cold, does the cough usually last more than 10days? ("Persistent cough")<br><br>**Assessed blind to outcome:**<br>Not reported<br><br>**Handling in the model:**<br>Used as binary variables: Yes/No	**Participants:**<br>Secondary care: 211<br>Primary care: 4280<br><br>**Events per variable:**<br>Secondary care: 95 / 4 = 23.75<br><br>**Participants with any missing data:**<br>Secondary care: 12<br>Primary care: 319<br><br>**Methods for handling missing data:**<br>Not reported for primary or secondary care.<br><br>**Model building**<br><br>**Modelling method:**<br>Logistic regression<br><br>**Model assumptions met:**<br>Not reported<br><br>**Selection of predictors:**<br>Not clear - they only use 4 questions from the survey - why they choose these is not reported.<br><br>They state: (in the abstract) "Four questions on the survey were shown to be sensitive and specific for asthma."<br><br>**Shrinkage of predictor weights:**<br>Not reported	**Final model presentation:**<br><br>In secondary care:<br>Wheeze<br>Nocturnal cough<br>Exercise symptoms<br>Persistent cough<br>Any of 4 symptoms<br><br>**Performance measures reported:**<br><br>In secondary care:<br>Any of 4 symptoms<br>Sensitivity: 100 (94 to 100)<br>Specificity: 55 (45 to 66)<br><br>**Model Evaluation**<br><br>**Internal validation:**<br>No internal validation reported<br><br>*Method used:* N/A<br>*Number in validation sample*: N/A<br>*Number with outcome:* N/A<br><br>**External validation:** No<br><br>Note: They report how the four questions relate in a different (primary care) sample - but do not report any measures of performance from this sample.<br><br>*If externally validated, was the model adjusted?* N/A	**COI:** Not reported.<br>Supported by a grant from the Patrick and Catherine Weldon Donaghue Medical Research Foundation.<br><br>**Strengths:**<br>Primary and secondary care populations included.<br><br>**Limitations:**<br>Development of model is unclear. Reporting of the performance of the model is also poor.<br><br>Inclusion of children below the age of 5 years.<br><br>**Generalisability:**<br>“In almost any population, a negative response to all 4 questions will mean that the child is very unlikely to have asthma.”

Study	Study population	Model development			Model performance and Evaluation	General information
		**Outcome**	**Predictors**	**Sample**		
**Hirsch 2001 & 2004**	**Design:**<br>Cross sectional survey completed at four time points (1993, 1995, 1999, 2001).<br><br>**Recruitment:**<br>*Method:* Initially postal questionnaire sent to all adults (≥16 years) registered at two GP practices.<br><br>Then selected individuals to achieve an asthma enriched sample. Participants were stratified for the likelihood they would have asthma based on responses to six questions.<br><br>Individuals from one practice were selected from each stratum to provide a stratified sample with equal numbers of asthma / no asthma who then attended for clinical assessment.	**Definition:**<br>Probability of asthma:<br><50%, 50-90%, >90%<br><br>"Those reviewed individuals in whom the consensus estimate of probability of asthma was 50% or more were designated clinically asthmatic and the remainder were designated clinically non-asthmatic."<br><br>**Measured:**<br>3 experts were provided with information from the clinical assessment (not the questionnaire data) and asked to categorise each patient into one of the three probability categories<br><br>Information collected:<br>• full history<br>• physical examination<br>• spirometry<br>• reversibility to beta-2 agonists<br>• bronchial challenge with histamine<br>• electronic peak flow diaries<br>• skin prick testing to five allergens.	**Description:**<br>Demographic information and questions from the survey<br><br>1. Age (16 - 34 years)<br>2. Age (35 - 54 years)<br>3. Age (55 - 74 years)<br>4. Sex<br>5. Wheeze in last 12 months<br>6. Breathless whilst wheezing<br>7. Wheeze in absence of cold<br>8. Woken by chest tightness in last 12 months<br>9. Woken by shortness of breath in last 12 months<br>10. Woken by night cough in last 12 months<br>11. Asthma attack last 12 months<br>12. Currently taking asthma medication<br>13. Family history of asthma<br>14. Hay fever/eczema ever<br>15. Smoker<br><br>**Assessed blind to outcome:**<br>Not reported	**Participants:**<br>1995 data (derivation):<br>10429 sent a postal survey<br>7582 responded (72.7%)<br>420 selected for clinical assessment<br>201 attended clinical assess (48%)<br><br>**Events per variable:**<br>84/15 = 5.6<br><br>**Missing data:**<br>180 participant's data used in analysis<br>21 had incomplete survey data.<br><br>**Participants with any missing data:**<br>21<br><br>**Methods for handling missing data:**<br>Excluded from modelling.	**Final model presentation:**<br>Age (16 – 34 years) 3<br>Age (35 – 54 years) 2<br>Age (55 – 74 years) 1<br>Sex (Male) 1<br>Wheeze in last 12 months 3<br>Breathless whilst wheezing 0<br>Wheeze in absence of cold -1<br>Woken by chest tightness in last 12 months 1<br>Woken by shortness of breath in last 12 months 0<br>Woken by night cough in last 12 months 0<br>Asthma attack in last 12 months 2<br>Currently taking asthma medication 1<br>Family history of asthma 1<br>Hayfever/eczema ever 1<br>Smoker -1<br><br>**Performance measures reported:**<br>Not reported	**COI:** None declared.<br><br>**Strengths:**<br>"the regression coefficients were not based on a single model but were the average of several representative models"<br><br>"an independent validation set comprising examples from the higher scores likely to be of interest when targeting diagnostic examinations, along with a small number of respondents believed to have a low probability of asthma."<br><br>**Limitations:**<br>Different outcome measures used in derivation and validation.
				**Model building**	**Model Evaluation**	
	Setting: General Practice.<br><br>*Location:* Wythenshawe, Manchester, UK.<br><br>**Eligibility:**<br>*Inclusion:* For the postal questionnaire - all adults registered at two GP practices.<br><br>Clinical evaluation of patients was drawn from the responders<br><br>*Exclusion:* Age < 16 years.<br><br>**Study Dates:** Derivation occurred in the 1995 dataset.<br><br>Validation in the 2001 dataset.	**Type:** Expert decision<br><br>**Same for all participants:** Yes<br><br>**Blind to predictors:** Probably Yes. Blind to the questionnaire responses<br><br>**Timing:** Data used from 1995 and 2001. There was a time lag between questionnaire response and clinical testing but duration not reported.	**Handling in the model:**<br>All variables were categorised:<br><br>Age (16 - 34 | 35 - 54 | 55 - 74 | ≥ 75)<br>Sex (Male/Female)<br>Survey questions: (Yes/No)	**Modelling method:**<br>Logistic regression<br><br>**Model assumptions met:** Not reported<br><br>**Selection of predictors:**<br>11 of the 12 survey questions included. Do not include household members smoking but this is not reported.<br><br>"The 4th age category (≥ 75) years is excluded from the model because it is dependent on the other 3 [age categories]"<br><br>**Shrinkage of predictor weights:**<br>"several candidate logistic regression models were produced and their respective coefficients averaged. These average values were rounded to the nearest whole number to give the question weights prior to summing to produce the weighted scores."	**Internal validation:** Yes<br><br>*Method used:* cross-validation technique with "several candidate logistic regression models" produced and the resulting coefficients averaged.<br><br>*Number in validation sample*: 180<br>*Number with outcome:* Not stated.<br><br>*If externally validated, was the model adjusted?* N/A	**Generalisability:**<br>Model not evaluated in other locations.<br><br>**Comments:**<br>Further analyses of the scoring system are completed in two related datasets. However, both datasets use different methods to measure the outcome, severely limiting these analyses as any difference in model performance could relate to the alternate outcome measure.

Study	Study population	Model development			Model performance and Evaluation	General information
		Outcome	Predictors	Sample		
**Lim 2015**	**Design:**<br>Cohort (? retrospective)<br><br>**Recruitment:**<br>*Method:* Participants were referred from other primary physicians or visited to pulmonary department by themselves without consultations.<br><br>*Setting:* Out-patient department<br><br>*Location:* South Korea<br><br>**Eligibility:**<br>*Inclusion:* "patients visiting the outpatient department with varied respiratory symptoms suggesting asthma, such as dyspnea, chronic cough, chest tightness and wheezing."<br><br>*Exclusion:*<br>1) current diagnosis of pneumonia, emphysema, tuberculosis or other lower respiratory tract diseases, and infections of the ear, sinus, or upper respiratory tract diseases<br>2) uncontrolled cardiovascular diseases, malignancy immunosuppressive diseases<br>3) patients hospitalized within 3 months due to other respiratory diseases<br>4) pregnant and breastfeeding women, and patients under 18 years old.<br><br>Subjects with other lung diseases including pneumonia, emphysema, tuberculosis, interstitial lung disease were excluded by radiologic examinations.<br><br>**Study Dates:** Not reported.	**Definition:** Asthma / No asthma<br><br>**Measured:**<br>"Participants were classified as asthmatics if the subjects were matched to the following criteria: 20% decrease in forced expiratory volume in 1 second (FEV1) with a dose of <16 mg/mL inhaled methacholine."<br><br>but also<br><br>"Patients were diagnosed with asthma if their answers to the questionnaire suggested it and the methacholine bronchial provocation test was positive."<br><br>**Type:** Unclear if single or combined outcome due to ambiguous reporting.<br><br>**Same for all participants:** Yes<br><br>**Blind to predictors:** Unclear<br><br>**Timing:** Unclear.<br>"At the first visit" is stated but no other visits reported.	**Description:**<br>5 questions "answered with the help of nurses or a physician."<br><br>Q1. Has the patient had an attack of wheezing?<br>Q2. Does the patient have wheeze or dyspnea after exercise?<br>Q3. Does the patient have a troublesome cough at night?<br>Q4. Did the patient's cold take more than 10 days to clear up?<br>Q5. Did the patient experience wheezing, chest tightness, or cough after exposure to airborne allergens or pollutants?<br><br>**Assessed blind to outcome:**<br>Not reported.<br><br>**Handling in the model:**<br>All responses were binary	**Participants:** 680<br><br>**Events per variable:** 164 / 5 = 32.8<br><br>**Missing data:** Not reported<br><br>**Participants with any missing data:**<br>Not reported<br><br>**Methods for handling missing data:**<br>Not reported	**Final model presentation:**<br>Each symptom is scored 1.<br><br>Attack of wheezing 1<br>Wheeze/dyspnea after exercise 1<br>Cough at night 1<br>Cold for more than 10 days 1<br>Allergens/pollutants causing symptoms 1<br><br>**Performance measures reported:**<br>Symptom score:<br>≥1 Sens 98.4% Spec 9.4%<br>≥2 Sens 86.3% Spec 20.4%<br>≥3 Sens 68.5% Spec 48.0%<br>≥4 Sens 39.5% Spec 74.6%<br>≥5 Sens 18.5% Spec 91.9%<br><br>The diagnostic value of the questionnaire was evaluated by ROC analysis. The AUC of the ROC curve was 0.610 ± 0.029"	**COI:** None declared.<br><br>**Strengths:**<br>"elucidate the clinical validity of a selectively chosen questions recommended by GINA for diagnosing asthma in the general adult population"<br><br>**Limitations:**<br>"no healthy control group"<br><br>No validation.<br><br>The recruiting hospital was in a city with "relatively severe" air pollution.<br><br>**Generalisability:**<br>Lack of validation limits generalisability.<br><br>**Comments**<br>The baseline characteristics between asthma and non-asthma groups was comparable except BMI which was higher in the asthma group.
				**Model building**	**Model Evaluation**	
				**Modelling method:**<br>Multivariate logistic regression<br><br>**Model assumptions met:**<br>Not reported<br><br>**Selection of predictors:**<br>All candidate predictors were included and kept in the model.<br><br>**Shrinkage of predictor weights:** N/A	**Internal validation:**<br>No internal validation reported<br><br>*Method used:* N/A<br>*Number in validation sample*: N/A<br>*Number with outcome:* N/A<br><br>**External validation:**<br>No external validation reported<br><br>*If externally validated, was the model adjusted?* N/A	

Study	Study population	Model development			Model performance and Evaluation	General information
		Outcome	Predictors	Sample		
**Metting 2016**	**Design:**<br>Retrospective cohort<br><br>**Recruitment:**<br>*Method:*<br>Retrospectively looked at records of participants who were referred to the asthma/COPD referral service by their GP for diagnostic assessment.<br><br>*Setting:*<br>Asthma / COPD referral center (interface between primary and secondary care)<br><br>*Location:*<br>Groningen, Netherlands<br><br>**Eligibility:**<br>*Inclusion:* Aged 15 or over and presenting with a respiratory complaint.<br><br>*Exclusion:* Participant unable to perform spirometry. Any participant with missing data.<br><br>**Study Dates:**<br>2007 to 2012<br><br>**Comment**: Only included data from experienced pulmonologists who had seen >300 patients in the referral service.	**Definition:**<br>Asthma, COPD<br><br>**Measured:**<br>Each patient was diagnosed by an experienced pulmonologist (n=10) using spirometry and history data provided to them i.e. didn't necessarily see the patient.<br><br>**Type:** Single endpoint<br><br>**Same for all participants:** Yes<br><br>**Blind to predictors:** No, candidate predictors informed the outcome.<br><br>**Candidate predictors part of the outcome**: No<br><br>**Timing:** Not reported.	**Description:**<br>*Participant characteristics*:<br>Age<br>Sex<br>Age of onset respiratory symptoms<br>Exacerbations<br>Allergy (No allergy / ≥ 1 allergy)<br>Current medication<br>Occupation (risk present / absent)<br>Smoking (Never / Ever)<br>Family history (No or unknown / positive)<br>Hyperreactivity<br><br>*Patient Reported Outcomes:*<br>Asthma Control Questionnaire (ACQ)<br>6 Questions<br>Clinical COPD Questionnaire (CCQ)<br>10 Questions<br><br>Spirometry:<br>Post bronchodilator FEV1(% predicted)<br>FVC (% predicted)<br>FEV1/FVC<br>Reversibility % (400 mcg salbutamol)<br><br>**Assessed blind to outcome:** Yes<br><br>**Handling in the model:**<br>These variables were transformed:<br>*Patient characteristics*<br>Age years <55 / ≥55<br>Age of onset years <38 / ≥38<br>BMI kg/m^2 <22 / ≥22 and <36 / ≥36<br>Allergy total: No allergy / ≥1 allergy<br>Hyperreactivity: No hyperreactivity / ≥1 hyperreactivity<br><br>ACQ1: 0 or 1 / ≥2<br>ACQ total, ACQ2, ACQ4, ACQ5, ACQ6: 0 / ≥1<br>CCQ subscale mental, CCQ1, CCQ2, CCQ4: 0 / ≥1<br>CCQ subscale symptoms, CCQ6: 0 or 1 / ≥2<br>CCQ7 <6 / ≥6<br><br>*Spirometry results*<br>FEV1 % predicted <78 / ≥78 and <92 / ≥92 and <102 / ≥102<br>FVC % predicted <81 / ≥81<br>Reversibility % <7 / ≥7	**Participants:**<br>10,058<br>9,297 with complete data<br>4125 with asthma outcome<br><br>**Events per variable:**<br>Minimum number of patients in a child leaf was 94 (>1% of total number of patients)<br><br>**Missing data:**<br>*Participants with any missing data:* 761<br>626 spirometry not possible<br>135 data missing at random<br><br>*Participants with missing data for each predictor:* 105 had data missing for Allergy<br><br>**Methods for handling missing data:**<br>Excluded from analysis.<br><br>**Model building**<br><br>**Modelling method:**<br>Chi-squared Automatic Interaction Detection (CHAID)<br><br>**Model assumptions met:**<br>Not reported<br><br>**Selection of predictors:**<br>*Before modelling:* "included all individual questions from the ACQ and CCQ and the total score on each questionnaire"<br><br>*During modelling:* Not clear<br><br>**Shrinkage of predictor weights:**<br>Bonferroni correction applied to correct for overstating of the significance level caused by multiple comparisons.	**Final model presentation:**<br>*6 branches (combinations) led to asthma*:<br><br>1) FEV1/FVC⩾70% pred, Onset<38years ⩾1allergy, Reversibility <7%<br><br>2) FEV1/FVC⩾70% pred, Onset<38years ⩾1 allergy, Reversibility ⩾7%<br><br>3) FEV1/FVC⩾70% pred, Onset<38years No allergy, Wheezing<br><br>4) FEV1/FVC⩾70% pred, Onset<38years ⩾1 allergy, Reversibility <7%<br><br>5) FEV1/FVC⩾70% pred, Onset<38years 1 allergy, Reversibility ⩾7%<br><br>6) FEV1/FVC <70% pred, Never smoked<br><br>**Performance measures (calculated)**<br>Decision tree (asthma) vs Pulmonologist<br><br>**Full / Simple / Ext validated**<br>Sens 0.79 / 0.72 / 0.78<br>Spec 0.75 / 0.79 / 0.60<br>PPV 0.72 / 0.73 / 0.35<br>NPV 0.82 / 0.78 / 0.91<br><br>**Model Evaluation**<br><br>**Internal validation:** Yes<br>*Method used:* 10-fold cross validation<br><br>*Number in validation sample*: Divided into 10 mutually exclusive subsets<br><br>*Number with outcome:* 9297<br><br>**External validation:** Yes Geographical validation - Rotterdam Asthma/COPD referral service.<br><br>*If externally validated, was the model adjusted?* Excluded FEV1 post bronchodilator to enhance applicability.	**COI:** Dr. Kocks grants from Zorgdraad foundation, during the conduct of the study; personal fees from GSK, outside the submitted work Dr. in 't Veen reports grants from Novartis, during the conduct of the study; grants from Picasso for COPD, grants from Astra Zeneca, outside the submitted work.<br><br>**Strengths:**<br>CHAID at least as good as logistic regression but easier to interpret.<br><br>Transparency they report is favoured by clinicians.<br><br>**Limitations:**<br>31% of patients could not be diagnosed correctly in comparison to the reference standard. Perhaps due in part to diagnostic variation between pulmonologists.<br><br>Absence of gold standard reference test - authors argue that pulmonologist diagnosis is most accurate.<br><br>Unable to differentiate patients with or without disease: "no disease", "indication of restriction" and "diagnosis unclear" are all combined. This was a small group n=709<br><br>**Generalisability:**<br>Have externally validated within a similar Netherlands asthma COPD referral service.

Study	Study population	Model development: Outcome	Model development: Predictors	Model development: Sample	Model performance and Evaluation	General information
**Schneider 2015**	**Design:**<br>Prospective cohort<br><br>**Recruitment:**<br>*Method:* Consecutively recruited patients presenting with respiratory symptoms.<br><br>*Setting:*<br>1) 10 GP practices<br>2) 5 private respiratory physician practices (Individuals in Germany can present direct to private physician)<br><br>*Location:*<br>1) Heidelberg, Germany<br>2) Bavaria, Germany<br><br>**Eligibility:**<br>*Inclusion:*<br>Dyspnea, cough or expectoration for more than 2 months<br><br>*Exclusion:*<br>Respiratory tract infections 6 weeks prior to evaluation.<br><br>Previous diagnosis of Obstructive Airways Disease (i.e. asthma/COPD/ACOS)<br><br>Untreated hyperthreosis, unstable CAD, cardiac arrhythmia<br><br>Pregnant<br><br>**Study Dates:**<br>1) Feb 2006 to June 2007<br>2) June 2010 to October 2011	**Definition:**<br>Asthma / COPD /<br>Asthma COPD Overlap (ACOS)<br><br>**Measured:**<br>Outcome assessed by a respiratory physician based on history, exam and either:<br><br>I) Positive Reversibility: FEV1/FVC <0.70 pre-bronchodilator, with reversibility of 12% and 200ml and lung function returned to the predicted normal range<br><br>II) Bronchial provocation leading to a 20% fall in FEV1 from baseline or a doubling of airway resistance to over 2.0kPa.<br><br>**Type:** Combined endpoint<br><br>**Same for all participants:**<br>No. 229 diagnosed with asthma<br>23 by reversibility<br>206 by bronchial provocation.<br><br>**Blind to predictors:**<br>Blind to FeNO. Not clear if blind to other candidate predictors.<br><br>**Timing:** Not clear if all tests completed on same day.	**Description:**<br><br>1) FeNO<br>*Questionnaire response*<br>2) Age<br>3) Sex<br>4) Breathlessness<br>5) Breathlessness on exertion<br>6) Wheezing ever<br>7) Cough<br>8) Respiratory tract infections<br>9) Expectoration<br>10) Woken by chest tightness<br>11) Woken by breathlessness<br>12) Nasal allergies<br>13) Medication for asthma<br>14) Current smoker<br>15) Ever smoked<br>16) Pack years of smoking<br><br>**Assessed blind to outcome:**<br>FeNO was. Otherwise unclear.<br><br>**Handling in the model:**<br>FeNO modelled as continuous and binary variable at different cut points.<br><br>Most questionnaire responses were categorical. Age and pack years smoking were continuous variables.	**Participants:**<br>560: Total in the combined samples<br>229: Total with asthma<br><br>**Events per variable:**<br>Paper states <10.<br><br>**Missing data:**<br>7 didn't have the outcome.<br><br>**Participants with any missing data:**<br>*Missing outcome: 7*<br>*Missing data for each predictor*: 81<br><br>**Methods for handling missing data:**<br>7 without outcome were excluded.<br><br>**Model building**<br><br>**Modelling method:**<br>Multiple logistic regression<br><br>**Model assumptions met:**<br>Not reported<br><br>**Selection of predictors:**<br>*Before modelling:* Only included the variables that were significant (p<0.05) following univariate analysis of each predictor variable with the outcome.<br><br>*During modelling:* Backward elimination with p>0.1 for exclusion was performed with the selected variables.<br><br>**Shrinkage of predictor weights:**<br>Not reported	**Final model presentation:**<br><br>*Model 1: GP*<br>FeNO, Age, medication, infection, cough<br><br>*Model 2: Private practice*<br>FeNO, wheezing, allergic rhinitis<br><br>*Model 3: Combined*<br>FeNO, Age, wheezing, allergic rhinitis AND medication, allergic rhinitis, infection<br><br>**Performance measures:**<br><br>Area Under the ROC curve<br>*Model 1:* 0.817 (0.745 to 0.889)<br>*n=129 (31 patients missing)*<br><br>*Model 2: 0.754 (0.703 to 0.806)*<br>*n=369 (24 missing)*<br><br>*Model 3:* 0.753 (0.707 to 0.789)<br>*n=472 (81 missing)*<br><br>**Model Evaluation**<br><br>**Internal validation:**<br>No internal validation reported<br><br>*Method used:* N/A<br>*Number in validation sample*: N/A<br>*Number with outcome:* N/A<br><br>**External validation:**<br>No external validation reported<br><br>*If externally validated, was the model adjusted?* N/A	**COI:** None declared.<br><br>**Strengths:**<br>"The strength of both settings was that only diagnostically naive patients presenting for the first time for diagnostic investigation were included."<br><br>**Limitations:**<br>No validation.<br><br>Participants included from two different settings<br><br>"participating patients had to travel to the lung function laboratory...which might have unintentionally caused a selection of patients with a higher probability and/or severity of disease."<br><br>**Generalisability:**<br>"decided to pool the data of both patient samples, because the clinical setting had only a minor influence on the sensitivities of the various cut-off points of FENO. As a result, the final model fitted well with the established clinical decision rules used by many physicians and led to a more conservative interpretation of the FENO measurements."<br><br>**Comments:**<br>Alternative presentation of model(s) as online calculator available:<br>http://bmjopen.bmj.com/content/suppl/2015/11/24/bmjopen-2015-009676.DC1

<table>
<tr><th rowspan="2">Study</th><th rowspan="2">Study population</th><th colspan="3">Model development</th><th rowspan="2">Model performance and Evaluation</th><th rowspan="2">General information</th></tr>
<tr><th>Outcome</th><th>Predictors</th><th>Sample</th></tr>
<tr>
<td rowspan="3">Tomita 2013</td>
<td rowspan="3">Design:
Prospective cohort

Recruitment:
Method:
Adults presenting to the clinic were referred to the study by physician

Setting:
Adult out-patient clinic

Location:
Kinki university hospital, Osaka, Japan

Eligibility:
Inclusion:
Adults presenting for the first time with non-specific respiratory symptoms.

Exclusion:
1) pregnant or breastfeeding
2) current diagnosis of: pneumonia/ pneumothorax atelectasis/ chronic bronchitis/ pulmonary fibrotic disease/ any other lower respiratory abnormalities
3) current inhaled/ oral steroids, beta-blockers or ACE Inhibitor medication
4) chest pain or haemosputum,
5) Abnormalities on X-Ray

Study Dates:
Jan 2008 - Sept 2011</td>
<td rowspan="3">Definition:
Asthma / no asthma
Measured:
Physician decision based on:
symptom history
AND
positive bronchodilator reversibility
OR
positive bronchial provocation

Type: Combined endpoint

Same for all participants:
Yes - everyone underwent reversibility and methacholine bronchial challenge

Blind to predictors:
Lung function and FeNO were measured blind to the interview and Asthma Screening Questionnaire (ASQ)

Timing:
1st visit: Interview/ physical exam/ASQ/ Lung function/FeNO blood samples.

2nd visit: Methacholine challenge test (performed within 8 weeks of first test)</td>
<td rowspan="3">Description:
Demographics:
1. Sex
2. BMI
3. Age
Asthma Screening Questionnaire (ASQ):
4. Score of 4 or more
Symptoms and history
5. Ever experience of wheeze
6. Diurnal variation of symptoms
7. Recurrent episodes
8. Medical history of allergic disease (asthma/atopic dermatitis/allergic rhinitis)
9. Family history allergy
Examination:
10. Wheeze on auscultation
Blood tests:
11. Eosinophils
12. Basophils
13. Total and specific IgE
Investigations:
14. FEV/FVC<0.7
15. % predicted FEV1 <95%
16. Bronchodilator reversibility
17. FeNO

Assessed blind to outcome:
Lung function and FeNO were measured blind to the interview and ASQ

Handling in the model
Use symptoms and presence of wheeze in the multivariable model only therefore all were binary variables</td>
<td>Participants:
4129 enrolled
3316 excluded
194 declined
53 missed bronchial provocation
566 included

Events per variable: 22
367 classified “asthma”
17 potential predictors

Missing data:
Not reported

Participants with any missing data:
Not reported

Methods for handling missing data:
Not reported</td>
<td>Final model presentation:
Algorithm diagram:
1 = Diurnal variation
1 = History of allergy
2 = Recurrent symptom episodes
2 = Wheeze on auscultation

High probability: 3 or more (90% probability of asthma)
Intermediate probability: 1 or 2 (68% probability of asthma)
Low probability: 0 (31% probability of asthma)

Performance measures reported:

Score 1 or more:
Sensitivity 0.89, specificity 0.83
Score 2 or more:
Sensitivity 0.57, specificity 0.92
Score 3 or more:
Sensitivity 0.35, specificity 0.97
Score 4 or more:
Sensitivity 0.16, specificity 1.00</td>
<td rowspan="3">COI: “None of the authors has a financial relationship with a commercial entity that has an interest in the contents of this manuscript”

Strengths:
Present the CPM as a flow diagram.

Limitations:
No validation.

If asthma diagnosed at 1st visit patients started on inhaled steroids, before methacholine challenge test within 8 weeks.

Stringent exclusion criteria

Generalisability:
Acknowledge the need for validation

Comments:
In addition to the model described they demonstrate how the probability of asthma changes for each score in the presence of a positive test:

If score 1 or 2 but have FEV1/FVC <70% then probability of asthma increased from 68 to 93%

If score 0 but positive reversibility test then probability of asthma increased from 31 to 88%</td>
</tr>
<tr><th>Model building</th><th>Model Evaluation</th></tr>
<tr>
<td>Modelling method:
Logistic regression

Model assumptions met:
Not reported

Selection of predictors:
Before modelling: Only used symptoms and signs in the modelling.

During modelling: Kept all predictors in the model presented, yet not two non-associated predictors (family history allergy / wheezing as a symptom) not in the final algorithm diagram.

Shrinkage of predictor weights:
Integer scoring system based on odds ratio from the model</td>
<td>Internal validation:
No internal validation reported

Method used: N/A
Number in validation sample: N/A
Number with outcome: N/A

External validation:
No external validation reported

If externally validated, was the model adjusted? N/A</td>
</tr>
</table>
